# Achieving more with less: lessons from country-level analyses of caesarean delivery and perinatal outcomes in Europe

**DOI:** 10.1093/eurpub/ckac129.112

**Published:** 2022-10-25

**Authors:** J Zeitlin, L Mortensen, JG Nijhuis, A Recio Alcaide, P Velebil, V Tica, E Mierzejewska, K Klungsoyr, S Donati, A Macfarlane

**Affiliations:** 1 Obstetrical Perinatal and Paediatric Epidemiology Research, Université Paris Cité, Inserm, Paris, France; 2 Methods and Analysis, Statistics Denmark, Copenhagen, Denmark; 3 Department of Obstetrics and Gynaecology, GROW School for Oncology and Reproduction, University Maastricht, Maastricht, Netherlands; 4 Universidad de Alcalá, Alcalá, Spain; 5 Institute for the Care of Mother and Child, Prague, Czechia; 6 East European Institute for Reproductive Health, University, Constanta, Romania; 7 Institute of Mother and Child, Warsaw, Poland; 8 Medical Birth Registry of Norway, Norwegian Institute of Public Health, Bergen, Norway; 9 National Center for Disease Prevention & Health, Superiore di Sanità, Rome, Italy; 10 School of Health Sciences, University of London, London, UK

## Abstract

**Background:**

There is consensus that caesarean delivery (CD) is a lifesaving procedure for both mother and child in emergency situations and that CD without medical indication should be avoided. However, the rate that optimally balances the risks and benefits of CD is unresolved. In 1985, the World Health Organization concluded that the CD rate should be no more than 10-15%; subsequent reviews relating CD rates to infant mortality show no benefits at the country-level for rates higher than 15-19%. However, stillbirth has not been investigated because comparable international stillbirth data are not readily available.

**Methods:**

We conducted an ecological study in 25 European countries from 2015 to 2019 utilizing data from routine birth data sources aggregated using the Euro-Peristat PHIRI federated data analysis protocol. We assessed country-level associations between CD rates and perinatal outcomes (singleton preterm birth, stillbirth at ≤ 24 weeks’ gestational age, neonatal death) for all years using Pearson correlations, adjusted for clustering of years within country. Correlations were also estimated between linear trends over time in the indicators.

**Results:**

The median [range] of CD rates was 23.1% [16.2 to 56.9] in 21 participating countries, while these were 6.9% [5.3 to 11.9] for preterm birth, 3.3 per 1000 total births [1.8 to 7.6] for stillbirth and 1.9 per 1000 live births [0.7 to 6.1] for neonatal mortality. The CD rate was not associated with the stillbirth rate (cluster-adjusted rho: -.01, P=.94) or with the neonatal mortality rate (rho:.27, P=.27). However, there was a strong positive correlation with the preterm birth rate (rho:.81, P<.001). Results were similar in time trend analyses.

**Conclusions:**

Higher CD rates were not associated with lower stillbirth or neonatal mortality rates, but were strongly correlated with higher preterm birth rates. This study suggests no benefits and indicates potential harms for higher CD rates in Europe.

